# Malaria Vaccines and Global Equity: A Scoping Review of Current Progress and Future Directions

**DOI:** 10.3390/biomedicines13061270

**Published:** 2025-05-22

**Authors:** Rajesh Perumbilavil Kaithamanakallam, Tirath Patel, Bharati Balachandran, Neville Fernandez, Joseph Jillwin, Dharambir Kashyap, Aparna Shivaprasad, Uttam Udayan, Pragnesh Kalyandrug, Aakanksha Aakanksha, Prasanna Honnavar

**Affiliations:** 1 Department of Microbiology and Immunology, American University of Antigua College of Medicine, St. Johns 1451, Antigua and Barbuda; prajesh@auamed.net (R.P.K.); bharatib@auamed.net (B.B.); nfernandez@auamed.net (N.F.); jjillwin@auamed.net (J.J.); ashivaprasad@auamed.net (A.S.); uudayan@auamed.net (U.U.); 2 Basic Medical Science, American University of Antigua College of Medicine, St. Johns 1451, Antigua and Barbuda; tirathp@auamed.net (T.P.); pragneshk@auamed.net (P.K.); 3 Brown Center for Immunotherapy, Melvin and Bren Simon Comprehensive Cancer Center, Division of Hematology and Oncology, School of Medicine, Indiana University, Indianapolis, IN 46202, USA; dbir@iu.edu; 4 Internal Medicine, Tbilisi State Medical University, 33 Vazha Pshavela Avenue, Tbilisi 0159, Georgia; aakankshasaharan1@gmail.com

**Keywords:** malaria vaccines, malaria prophylaxis, malaria endemicity, global health equity, vaccine efficacy and implementation

## Abstract

The journey toward a viable malaria vaccine, initiated in 1965, reached a major milestone in 2021 with the WHO’s endorsement of RTS,S/AS01, a recombinant protein-based malaria vaccine. This progress continued with the 2023 approval of the R21/Matrix-M vaccine, which is more cost-effective, more potent due it is higher protein content, and easier to manufacture. Though these achievements signal hope, malaria’s intricate life cycle and its prevalence in underprivileged regions make vaccine development and equitable distribution challenging. This review explains the lifecycle of malaria and explores the evolution of various treatment strategies aimed at reducing malaria-related mortality. This scoping review aims to provide a comprehensive overview of malaria vaccines, examining their development, efficacy, safety, and implementation challenges. Using a structured literature search across PubMed, Web of Science, and Scopus, we identified key themes related to malaria vaccines trials, policy implications, and future research needs. Peer-reviewed publications on PubMed, Scopus, and Web of Science from 1970 to 2024 were searched without any limitations. Search and Boolean search terms were modified to include terms like “malaria vaccines”, “malaria vaccination”, “malaria immunisation”, “malaria immunisation AND malaria-endemic countries”, “malaria endemic low-income countries AND malaria control”, “malaria public health control”, “malaria chemoprophylaxis AND early diagnosis of malaria”, “screening for malaria”, and “laboratory diagnosis of malaria in endemic countries” in order to find pertinent studies. Preliminary insights suggest that although vaccines are crucial, comprehensive strategies involving health education, hygiene, and timely medical intervention remain essential to malaria control.

## 1. Introduction

Malaria is a major public health problem, with nearly 250 million cases reported annually. This represents about 0.35% of the global population. Therefore, malaria poses a significant public health challenge, particularly for children under five years of age and pregnant women. Approximately 90% of cases or more occurs in Africa, while the remaining are distributed across Southeast Asia and the Eastern Mediterranean Region. Malaria causes approximately 10% of all children’s deaths in Africa. In Africa, 38 countries are affected by malaria, including Nigeria (with the highest number of malaria cases worldwide), Ghana, Uganda, Tanzania, Burkina Faso, Mali, Niger, and the Democratic Republic of Congo. Southeast Asian and the Eastern Mediterranean region countries include India, Indonesia, and Pakistan. Middle East and North Africa are also at risk, including countries such as Iran, Yemen, and Afghanistan. Malaria causes nearly 600,000 deaths worldwide each year, which makes it a public health concern that requires immediate attention [1,2,3,4,5].

Five species of *Plasmodium* parasites are known to cause malaria in humans, including *Plasmodium falciparum*, *Plasmodium vivax*, *Plasmodium malariae, Plasmodium ovale*, and *Plasmodium knowlesi* (which can affect humans and other primates but is less common). *P. falciparum* and *P. vivax* pose a significant threat to the population. *P. vivax and P. falciparum* are associated with the greatest number of deaths, with their prevalence and impact varying by geographic region. Cerebral edema, which arises due to inflammation, can lead to coma or death of the infected person and is a complication of infection with *P. falciparum*. The inflammation is caused by the sequestration of the infected erythrocytes that usually adhere to the vascular endothelium and do not circulate freely in the blood.

Malaria is a complex and multifaceted mosquito-borne infectious disease. The disease can manifest with a wide range of symptoms, including fever, fatigue, headache, vomiting, chills, abdominal pain, malaise, confusion, night sweats, and diarrhea, typically appearing around one or two weeks after being bitten by an infected female *Anopheles* mosquito. The severity of these symptoms depends on the *Plasmodium* species, with *P. falciparum* being the most virulent and causing severe and life-threatening symptoms. Additionally, the infected person’s immune status level is crucial in the outcome of the severity of symptoms. Individuals who have been previously exposed to malaria or have some level of immunity experience milder symptoms or even asymptomatic infections.

Malaria pathogenesis begins when an infected *Anopheles* introduces *Plasmodium* into the host bloodstream, where it evades immune detection by hiding inside the erythrocytes. In detail, the cycle begins when an infected female *Anopheles* mosquito bites a human, releasing saliva with anticoagulant proteins into the bloodstream. Following this, the parasite successfully evades the host’s immune system by residing inside the erythrocytes, which provides a safe and nutrient-rich environment for its development and growth. Inside the erythrocytes, the parasite undergoes various stages of development, including the ring stage, the trophozoite stage, and the schizont stage. During this development process, the parasite undergoes various transformations, including releasing toxic substances such as hemozoin (a byproduct of hemoglobin breakdown). When these erythrocytes lyse and release merozoites, hemozoin is released into the bloodstream, triggering an inflammatory response that leads to fever, chills, and other symptoms.

Female *Anopheles* mosquitoes primarily transmit malaria through their bites. Once infected, the mosquito transmits the infective form of the parasite, namely, the sporozoite, through subsequent bites. Alternatively, malaria can be spread via blood transfusion, sharing contaminated needles, and from mother to fetus during pregnancy. However, malaria is not transmitted from person to person. The incubation periods for different species of malaria parasites vary. The incubation period of *P. falciparum* is 9–14 days, and that of *P. vivax* ranges from 12–18 days but can also occur months after exposure. The incubation period of *P. malariae* is 18–40 days, *P. ovale* is 18 days, and *P. knowlesi* is 9–12 days.

Malaria is a global health concern that impacts millions of people worldwide. Therefore, taking active measures to prevent its spread is very important. Various methods exist to prevent malarial infection, such as using insecticides, nets, mosquito repellents, appropriate clothing, vaccines, and more. However, specific preventive measures should be planned according to the region. One effective method of preventing malaria is using insecticide-treated mosquito nets to protect against mosquito bites. These long-lasting insecticidal nets (LLINs) are designed to remain effective for several years and decrease malaria transmission. These insecticidal nets are critical in endemic regions as they are cost-effective and ensure widespread coverage and accessibility [4,5,6]. Indoor residual spraying (IRS) is another intervention used to prevent malaria through the application of insecticides on the walls and surfaces of homes. It helps kill adult mosquitoes, thereby reducing the population of malaria vectors and helping reduce the transmission of malaria [7]. Various insecticides are used, including pyrethroids, carbamates, organophosphates, etc. A study conducted in Uganda evaluated the effectiveness of these preventive measures and found that the combination of LLINs and IRS is more effective in reducing malaria burden, especially in high transmission areas. These findings show the importance of implementing comprehensive and tailored malaria prevention strategies in different regions [8].

The World Health Organization (WHO) recommends sulfadoxine-pyrimethamine (SP-iPTi) as one of the interventions to prevent malaria in infants [9]. Few studies have been conducted regarding the effectives of this chemoprophylaxis [10]. In Nigeria, a randomized controlled trial (RCT) conducted to determine the efficacy of SP-IPTi in 2019 and 2021 revealed that although the antibiotic prophylaxis was well tolerated by the participants, it had no overall benefit. Additionally, the malaria parasite can also develop resistance to this intervention [11,12,13].

Another important measure to reduce the burden of malaria is the use of malaria vaccines. Vaccine development started around the 1940s and 1950s [14]. It involved the development of the first malaria vaccine using killed or attenuated *Plasmodium*. During the 1960s, the focus was on using whole-killed sporozoites as vaccines, with sporozoites being the parasite’s infecting form following the mosquito bite. Trials demonstrated that while these vaccines could induce some immune response, they were ineffective. The challenges included the parasite’s life cycle complexity and the need for multiple doses [15].

**Figure 1:**
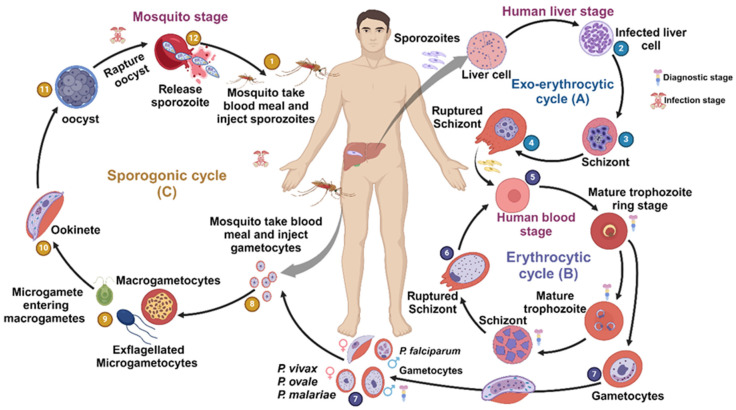
Life cycle of the malarial parasite.

The life cycle of the malarial parasite (Figure 1) involves the sporogony cycle in the definitive host the mosquito and the erythrocytic cycle in humans, the intermediate host. The infected definitive host inoculates the infecting form, namely, the sporozoites, into the intermediate host, namely, humans. The sporozoites infect the hepatocytes and evolve into schizonts. The schizonts rupture and release merozoites that can infect other hepatocytes or be released into the blood stream. In infections with *P. vivax* and *P. ovale*, a dormant stage termed hypnozoites can persist in the liver and cause relapses. After replication in the liver (exo-erythrocytic schizogony), the merozoites of the *Plasmodium* infect the intermediate host’s erythrocytes (erythrocytic schizogony). In the erythrocytes, the ring stage trophozoites mature into schizonts, which again rupture, releasing merozoites. These merozoites infect other red blood cells, thereby propagating the lifecycle. Few of the parasites differentiate into the sexual erythrocytic stage (gametocytes), which are ingested by the female anopheles mosquito.

The sporogony cycle starts with the ingested gametocytes, including male (microgametocytes) and female (macrogametocytes), in the mosquito’s gut. Zygotes are formed with the fusion of microgametes and macrogametes. The motile zygotes (ookinetes) invade the gut wall and develop into oocysts. The oocysts grow, rupture, and release sporozoites, which make their way to the mosquito’s salivary glands. Inoculation of the sporozoites into a new human host perpetuates the malaria life cycle (Figure 1).

From 1980s to the 1990s, molecular biology advances helped to identify specific antigens associated with the Plasmodium parasite, such as circumsporozoite protein (CSP) and merozoite surface proteins (MSP) [16,17,18]. Subunit vaccines developed using recombinant DNA technology targeting these specific antigens.

DNA vaccines emerged as a novel approach during the 1990s. This technology involved plasmid DNA to encode malaria antigens, allowing the host cells to produce the antigens and stimulate an immune response. These vaccines offered several advantages, such as ease of production, stability, and the potential to target multiple antigens simultaneously [19,20]. In the 2010s, mRNA vaccines began to gain traction. Studies focused on designing mRNA constructs that encode malaria antigens to induce strong immune responses. Currently, trials for mRNA malaria vaccines are ongoing with several candidates in different regions worldwide. Researchers are exploring self-amplifying RNA technology to improve vaccine response. Researchers are also exploring the potential of combining mRNA vaccines with other vaccine platforms to create a more robust immune response [21].

**Figure 2:**
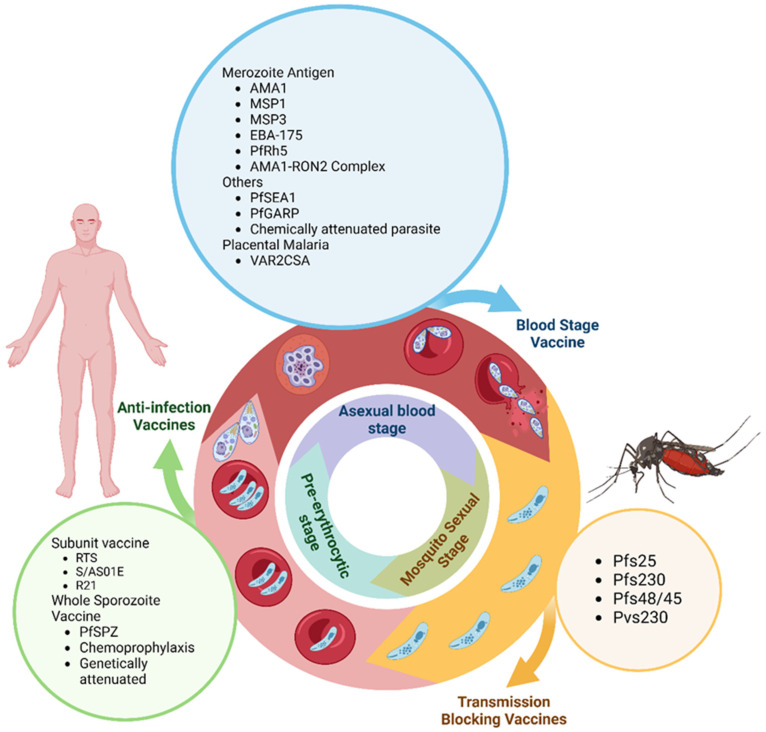
Malaria vaccines acting on different stages of the life cycle of the parasite [5].

Vaccination is observed to be the most efficient way to reduce the burden of malaria. Malaria vaccines target different stages of *Plasmodium*’s life cycle (Figure 2). Vaccines are mainly developed against *P. falciparum* and *P. vivax* species. Some main categories of vaccines include pre-erythrocytic vaccines, subunit vaccines (liver stage), erythrocytic vaccines (blood stage), transmission-blocking vaccines, viral vector vaccines, DNA vaccines, and mRNA vaccines [22,23,24,25,26].

## 2. Types of Malaria Vaccines

### 2.1. Pre-Erythrocytic Vaccines

Pre-erythrocytic vaccines prevent liver-stage infections by targeting the sporozoite stage (injected by the bite of an infected female *Anopheles* mosquito) before the parasite reaches the blood stream [27]. These vaccines can induce both antibody-mediated and cellular immune responses. Antibodies can neutralize various parasite proteins, such as the CSP, which prevent the parasite from entering the liver stage. The cellular immune response elicited by the vaccine helps generate CD8+ cells, which can recognize and kill cells infected with the parasite.

These pre-erythrocytic vaccines include RTS,S/AS01 (Mosquirix), which target the CSP of *P. falciparum* and induce immunity against the sporozoite stage. Many trials across different regions have reflected on the efficacy of RTS,S/AS01 vaccine [28]. RTS,S is a recombinant vaccine, composed of parasite’s CSP and the hepatitis B surface antigen (HBsAg). To enhance the immune response, this vaccine is formulated with two different adjuvant systems: one uses liposomes (AS01) and the other an oil-in-water emulsion (AS02). A randomized control trial (RCT) conducted with healthy children aged 5–17 months in Kenya, Kilifi, Korogwe, and Tanzania from 2007 to 2010 revealed that this vaccine protected against clinical malaria in children. After 15 months, vaccine efficacy was found to be 45.8%; antibody levels were also increased. Side effects associated with the vaccine include convulsions, pneumonia, and gastroenteritis [29].

A trial was conducted to assess the safety of the RTS,S/AS01 malaria vaccine in HIV-infected children residing in sub-Saharan Africa. The study was conducted at various sites, specifically Kenya, Mozambique, and Malawi. The study revealed that the vaccine had adverse events, but it was almost similar in both the HIV-infected children and non-HIV-infected controls. Various adverse events were frequently reported, such as gastroenteritis, pneumonia, malnutrition, anemia, and meningitis, mostly in children under the age of two. The study found that the vaccine has a lower immunogenic response in HIV-infected children, indicated by lower anti-CS antibody geometric mean concentrations (GMCs) in HIV-infected children as compared to HIV-negative controls. The results of the study showed that the vaccine is generally safe to be used in HIV-infected populations. Still, monitoring vaccine safety remains critical in populations with comorbidities like HIV and others residing in sub-Saharan regions [30].

Evaluation of the efficacy, safety, and immunogenicity of the RTS,S/AS01 malaria vaccine from 2009–2011 in children aged 5–17 months was the aim of a study performed in seven African countries. The results demonstrated an efficacy of 55.8% during the 12-month follow-up. The level of effectiveness was lower at the end of the follow-up compared to the efficacy after the vaccination. It was generally safe, but some severe events were also reported, and there was no reduction in overall mortality [31]. Another paper quoted this study and observed that the average number of cases prevented per 1000 vaccinated children ranged between 37 and 2365 across different regions. Incidence reduction was reduced with time, with a 68% reduction in clinical malaria during the first six months after vaccination, which decreased to 26% by months 13–18. The vaccine also showed a reduction in malaria episodes by 46% and a 41% reduction in malaria hospitalization. Besides reducing malaria cases, the vaccine also showed severe adverse events such as meningitis [32]. The randomized controlled trial conducted at 11 centers in seven countries, including Burkina Faso, Gabon, Ghana, Kenya, Tanzania, Malawi, and Mozambique, in sub-Saharan Africa between 2009 and 2014 to determine the safety of the RTS,S/AS01 malaria vaccine in infants and children was cited by another publication. It was documented that the vaccination resulted in various adverse events, especially in infants, including febrile convulsions, meningitis, cerebral malaria, rashes, and mucocutaneous lesions. Even though the benefit-risk balance was positive, these adverse effects demand further investigation and the development of safer vaccines [33].

Another example of a pre-erythrocytic vaccine is R21/Matrix-M, which is effective in various clinical trials. It is used in some African countries given its high efficacy and favorable safety. R21/Matrix-M is a recombinant protein vaccine comprising a fusion of HBsAg with *P. falciparum*’s CSP. Its design helps enhance the formation of virus-like particles, which are very important in eliciting a strong immune response. Novavax’s Matrix-M adjuvant technology significantly boosts the immune response and increases the vaccine’s efficacy. This vaccine has been through various clinical trials, including phase 3 trials involving around 4800 children across several African countries. It showed high efficacy rates, especially in seasonal malaria transmission areas [34].

PfSPZ vaccine is also a pre-erythrocytic vaccine. It uses live-attenuated *P. falciparum* sporozoites to induce antibody production (against CSP) and potent T cell responses. These irradiated parasites fail to complete liver-stage development, thereby preventing blood stage infection. It is a whole sporozoite vaccine containing irradiated sporozoites to prevent them from causing malaria.

Various trials have been conducted to determine the safety profile of this vaccine in the population at risk of malaria. One study was conducted in which participants were given vaccine doses from March 2017 to July 2017 and were observed after that to see the outcomes. It was observed that the adult population involved in this trial showed a reduced immune response compared to the children who participated. This study suggests that the longer exposure time to malaria in adults from Africa has potentially contributed to the lower immune response. The vaccine was found to be effective overall but was found to be associated with certain mild adverse events such as tachycardia, tachypnea, and bradycardia [35].

Another trial, conducted in Tanzania from 2015 to 2017, was designed to determine the safety, immunogenicity, and efficacy of the PfSPZ vaccine. This study found that increasing the dose of the vaccine increases the effectiveness, but only to a certain extent. The results showed that various doses of the vaccine result in different durations of protective effects with varying levels of immune response elicited [36].

### 2.2. Erythrocytic Vaccines

Erythrocytic vaccines target the merozoite stage of the *Plasmodium* parasite, which targets the red blood cells [37,38,39,40,41]. These vaccines target various antigens, and examples include apical membrane antigen 1 (AMA1), erythrocyte-binding antigen 175 (EBA-175), and MSPs. AMA1 targets proteins expressed on the surface of merozoites and is designed to block the invasion of red blood cells [42,43]. EBA-175 targets another protein called erythrocyte binding antigen 175, which is a protein that binds to glycophorin A (GPA), a glycoprotein on the surface of human erythrocytes [44]. Erythrocyte-binding antigen is essential for merozoite invasion of red blood cell. MSPs targeting vaccines are being developed to prevent the parasite from invading the red blood cells.

### 2.3. Liver-Stage Subunit Vaccines

Liver-stage vaccines are a key focus in malaria vaccine research. These vaccines target the liver stage of the parasite’s life cycle. The stage occurs after the mosquito’s bite, which results in sporozoites being injected into the human host’s body, which will migrate to the liver and develop into merozoites. As the liver stage is crucial for the transmission and pathogenesis of malaria, these vaccines aim to trigger immune responses that eliminate liver-stage parasite. Many vaccines use viral vectors such as adenovirus, stomatitis virus, and others to deliver *Plasmodium* antigens, generating the desired immune efficacy. A good example of this vaccine is the ChAd63-MVA vaccine, which targets thrombospondin-related adhesion protein (TRAP) [45,46].

### 2.4. Transmission-Blocking Vaccine

Transmission-blocking vaccines, such as Pfs25 and Pfs230, aim to interrupt the spread of the malarial parasites from humans to mosquitoes [47,48]. The Pfs25 vaccine targets certain proteins on the sexual stages of *Plasmodium* in the hindgut of mosquito which prevents the infectivity of the parasite [49].

### 2.5. Viral Vector Vaccines

Viral vector vaccines use genetically engineered viruses such as adenovirus, MVA, and others to deliver to deliver malarial antigens into host cells, where they stimulate robust CD8+ T cell responses and long-term memory immunity. ChAd63/MVA is one example, which uses chimpanzee adenovirus (ChAd63) and modified vaccinia Ankara (MVA) to deliver malarial antigens to induce strong cellular immunity against the parasite in the liver stage [46,50].

### 2.6. DNA and mRNA Vaccines

DNA vaccines use plasmid DNA to encode various types of malarial antigens. DNA vaccines are under investigation for different *Plasmodium* life stages. mRNA vaccines are a recently developed class of vaccines that uses messenger RNA to instruct cells to produce malaria antigens. DNA and RNA vaccines are undergoing various stages of research and development.

Various other diseases are common in areas that are at higher risk of malaria. One such disease is Ebola. Therefore, one study sought to determine whether an asymptomatic malaria infection causes any effects in the immune response to a two-dose Ebola vaccine regimen consisting of Ad26.ZEBOV and MVA-BN-Filo in adults as well as children. The results showed that the vaccine elicited an immune response effectively, even in asymptomatic malaria patients who participated in this study. This shows that it is unnecessary to screen for asymptomatic malaria prior to this immunization regimen [51].

All these trials showed the efficacy of vaccine use, which makes it essential to ensure that the population in malaria-endemic regions has enough knowledge about vaccines and is ready to get vaccinated against malaria. To determine this, a study was conducted in Nigeria involving caregivers of children under five. The data collected in this survey showed that the participants showed a high vaccine acceptance rate [52].

## 3. Methods

This review followed the established procedures outlined in the Preferred Reporting Items for Systematic Review and Meta-analysis (PRISMA). The PRISMA methodology (Preferred Reporting Items for Systematic reviews and Meta-Analyses) is a set of guidelines designed to enhance the transparency and completeness of reporting in systematic reviews and meta-analyses. This review has been registered with PROSPERO with registration number CRD42024588864.

**Figure 3:**
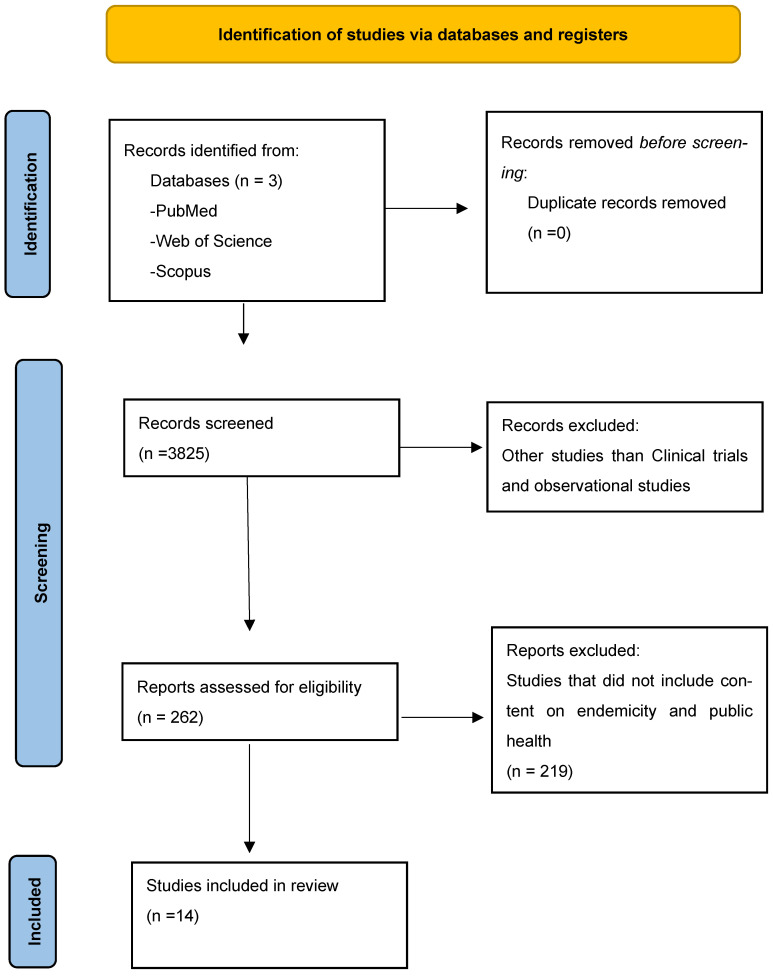
Prisma flow diagram.

Peer-reviewed publications on PubMed, Scopus, and Web of Science from 1970 to 2024 were searched without any limitations (Figure 3). Search and Boolean search terms were modified to include terms like “malaria vaccines”, “malaria vaccination”, “malaria immunisation”, “malaria immunisation AND malaria-endemic countries”, “malaria endemic low-income countries AND malaria control”, “malaria public health control”, “malaria chemoprophylaxis AND early diagnosis of malaria”, “screening for malaria”, and “laboratory diagnosis of malaria in endemic countries” in order to find pertinent studies. Only human studies and English-language publications were included. After conducting a detailed manual search of all potential research publications and international guidelines, a cross-search for additional relevant research was conducted. A structured data charting form was developed to extract information on vaccine type, trial outcomes, implementation barriers, and policy recommendations. Two independent researchers evaluated the published publications critically, and disagreements over interpretations were resolved through group discussions and involvement of a third reviewer when necessary.

### 3.1. Inclusion Criteria

This review contains RCTs, cohort studies, and case–control studies undertaken in malaria-endemic countries, with a focus on low-income settings. The study selection criteria covered all forms of healthcare support, such as chemoprophylaxis, vector control, and immunization, for human subjects of all ages. Studies assessing vaccine trial schedule, outcomes, and follow-up in the different populations were included in this review. Furthermore, the study articles included were suggestive of malaria vaccination with other malaria control techniques (e.g., chemoprophylaxis, vector control, early diagnosis, and treatment) to assure a longer treatment period. All the articles featured were written in English.

### 3.2. Exclusion Criteria

Research on vaccine development or technology alone, without considering the consequences for public health or the management of malaria in endemic areas, was not included in this review. Additionally, research that did not offer adequate information about the incidence, prevalence, or mortality rates of malaria in the community being studied was disregarded. Exclusion criteria also included research done on non-human populations, in non-malaria endemic areas, and without comparisons or control groups. Additionally, papers with insufficient data (studies lacking clear outcome measures, sample sizes, or relevant details on vaccine efficacy or safety) were not included in this review.

## 4. Discussion

This review highlights key advancements in malaria vaccines, including the transition from RTS,S/AS01 to R21/Matrix-M and growing interest in mRNA vaccine platforms. However, challenges remain in vaccine rollout, including supply chain constraints, acceptance in malaria-endemic regions, and long-term immunity concerns. Future research focus on optimizing vaccine schedules, integrating malaria vaccines with other public health interventions, and assessing cost-effectiveness in low-resource setting.

Malaria remains a growing global concern. Vaccines play a critical role in malaria control. One important type of vaccine is RTS,S/AS01 which has been widely used and strongly recommended by the WHO [32,53,54,55,56,57,58,59,60,61,62,63]. Summary table of the reviewed articles is given in Table 1. In a study performed by Agnandji et al., the researchers tried to understand the efficacy of the RTS,S/AS01 vaccine in seven African countries. The study had a sample size of 15,460 children and was divided into the RTS,S/AS01 or the non-malaria comparator (control) vaccine group. Out of the large sample, the first group of children included 600 patients who were in the age range of 5 to 17 months. Looking at the episodes to person year ratios, the study reported values of 0.32 and 0.55 for RTS,S/AS01 and control, respectively. The efficacy of RTS,S/AS01 was higher at 50.4% compared to the control [31].

A similar study by Olotu et al. evaluated RTS,S/AS01 efficacy in children from Tanzania and Kenya. The sample had 894 children between 5 and 17 months. The RTS,S/AS01 vaccinated group consisted of 447 patients, and the control group, vaccinated against rabies, had 447 patients as well. The vaccine efficacy (VE) values of the RTS,S/AS01 vaccine for children at 12 to 18 months are 39.2% (*p* = 0.0005) and 45.8% (*p* = 0.0004), respectively. Convulsions, pneumonia, and gastroenteritis are common adverse effects seen in the RTS,S/AS01 group [21]. Based on these two studies, the efficacy of RTS,S/AS01 seems to be pivotal in treating children, especially in African countries.

Despite RTS,S/AS01 being the current gold standard, new vaccines are being developed to improve efficacy and reduce side effects. For example, a study by Sirima et al. wanted to understand the effectiveness of whole sporozoite vaccines in Burkina Faso. The sample consisted of n = 80 patients. Here, the *P. falciparum* sporozoite vaccine (PfSPZ vaccine) group had n = 39 patients, and the normal saline control group had n = 41 patients. The study looked at both groups’ vaccine efficacy rates and anti-circumsporozoite antibodies. The vaccine efficacy rates at the 6- and 18-month points for the PfSPZ vaccine were 38% (*p* = 0.017) and 15% (0.078), respectively. The study also showed that the PfSPZ vaccine group had increased levels anti-circumsporozoite antibodies compared with the control group after two weeks.

Another study by Verma et al. attempted to test the effectiveness of the R21/Matrix-M malaria vaccine compared to RTS,S/AS01 (RTS,S) in four different African countries. The sample consisted of n = 4800 patients. For the children between the 5 and 17 months, the seasonal effectiveness was higher in the R21/Matrix-M malaria vaccine group. R21/Matrix-M showed 75% and 74% efficacy at 12 and 18 months, respectively. Finally, the study also reported that the anti-NANP antibody titers were more than doubled in the R21/Matrix-M malaria group than in the RTS,S/AS01 (RTS,S) group after the third dose [21]. Based on these two studies, the PfSPZ and R21/Matrix-M vaccines help prevent malaria endemic, along with the gold standard RTS,S/AS01 vaccine.

With vaccines being the primary focus to prevent the spread of malaria in the population, some standard community measures can provide additional support to avoid endemics [4,64,65,66,67,68]. For example, in a study by Katureebe et al., the researchers wanted to test the use of LLIN and IRS of insecticides in three Uganda locations. The sample sizes in each of these locations were as follows: Walukuba (n = 42,833), Kihihi (n = 28,790), and Nagongera (n = 38,690). The study looked at three different factors in these areas: the incidence of malaria (episodes per person per year (PPY), human biting rates (HBR), and test positivity rate (TPR). In Walukuba, using LLIN, there were no changes in all three parameters. Kihihi showed a decrease in malaria incidence (1.77 pre-intervention versus 1.89 post-intervention; aRR = 0.65, 95% CI 0.43–0.98, *p* = 0.04) but no changes in TPR or HBR. Finally, in Nagongera, there was a reduction in TPR (45.3% pre-intervention versus 36.5% post-intervention; aRR = 0.82, 95% CI 0.76–0.88, *p* < 0.001) but no changes in the incidence and HBR [31]. Based on these results, we can see that measures such LLIN can provide additional support along with vaccines to pause and prevent malaria endemics.

The future development of vaccines should prioritize improving antigen design and adjuvant systems to enhance efficacy along with minimizing adverse effects. Understanding the underlying mechanisms behind adverse events (meningitis, gastroenteritis, etc.), whether due to vaccine components, immune overreactions, or co-incidental infections, requires post-vaccination surveillance. The use of pharmacogenomic screening and targeted vaccine delivery could help in reducing complications.

## 5. Conclusions, Safety Concerns, and Limitations

Vaccines certainly have a major stake in preventing malaria morbidity and mortality in malaria-endemic countries. This scoping review highlights the progress in malaria vaccine development while identifying critical gaps in vaccine implementation, acceptance, and long-term protection. Although the studies mentioned were focused mainly on vaccine usage and efficacy, more RCT studies especially with phase 4 components should be performed. This would help any adverse effects seen in the patient pool and give researchers the data to further research and modify the vaccines. The limitations of our scoping review include potential publication bias, exclusion of non-English studies, and the lack of qualitative synthesis. Further studies should explore innovative strategies and real-world effectiveness in endemic settings to support global malaria elimination efforts.

## Figures and Tables

**Table 1:** Summary table of the reviewed articles.

S. No	Reference	Year of Publication	Purpose of Research	Number of Patients Initially Assigned	Result
1	Agnandji et al. [31]	17 November 2011	To test the efficacy of the phase III malaria vaccine RTS,S/AS01 in seven African countries	N = 15,460 children	In the study, the first 600 children were from the 5 to 17 months category. There were 2 groups where one received the RTS,S/AS01 vaccine and the other got the non-malaria comparator (control) vaccine. The study had looked at vaccine episode to person-year ratios of RTS,S/AS01 and control, which showed values of 0.32 and 0.55, respectively. The study revealed that the efficacy RTS,S/AS01 was 50.4% (95% confidence interval [CI], 45.8 to 54.6) in comparison to the control.
2	Agnandji et al. [32]	29 July 2014	Observing the effects of phase 3 RTS,S/AS01 after 18 months in children at 11 African sites	N = 6537 infants (6–12 weeks), N = 8923 children (15 to 17 months)	The study was looking at the vaccine efficacy in both groups with either the RTS,S/AS01 or the comparator (control vaccine). The vaccine efficacy rate (VE) for the children group for the first 18 months was 46% (95% CI 42% to 50%) (range 40% to 77%; VE, *p* = 0.01 across all sites). The VE for the infant group was 27% (95% CI 20% to 32%, per protocol; 27% [95% CI 21% to 33%]). The children group had better protection against severe malaria, malaria hospitalization, and all cause hospitalization than the infant group. One big adverse condition with this vaccine was meningitis. In the RTS,S/AS01 vaccine groups, there were 16/5949 and 9/4358 adverse events in the children and infant groups, respectively.
3	Tsoumani et al. [63]	4 September 2023	Testing the use of mRNA-based vaccines to treat malaria		Previously, mRNA vaccines have been helpful in treating various infections such as COVID-19. In terms of malaria, mRNA amplifying vaccines have been showing clinical efficacy. The vaccine targets *Plasmodium* macrophage migration inhibitory factor (PMIF) released from the parasites. The study has also shown that vaccines with antigens containing Pfs25 and *Pf*CSP had generated a stronger immune response. These mRNA-lipid nanoparticles (LNP) vaccines prevent the parasite from moving into red blood cells.
4	Sirima et al. [35]	7 December 2022	Understand vaccine efficacy of whole sporozoite vaccine in the area of Balonghin, Burkina Faso	N = 80 patients, n = 39 *Plasmodium falciparum* sporozoite vaccine (PfSPZ vaccine), n = 41 normal saline (control)	The adults had been given three doses of either vaccine. For the PfSPZ vaccine, the vaccine efficacy (1 − risk ratio; primary VE endpoint) was 38% at 6 months (*p* = 0.017) and 15% at 18 months (0.078). After a 2-week period from the vaccine administration, patients who took PfSPZ had shown more antibodies to *P. falciparum* circumsporozoite proteins than the control group.
5	Olotu et al. [29]	11 February 2011	Studying the efficacy of RTS,S/AS01 in children 5–17 months in Kenya and Tanzania	N = 894, n = 447 (RTS,S/AS01), n = 447 (rabies vaccinated)	The study focused on determining the estimated duration that the anti circumsporozoite antibodies lasted with the RTS,S/AS01 vaccine. The vaccine efficacy rates (VEs) at 12 and 18 months were 39.2% (95% CI 19.5–54.1, *p* = 0.0005) and 45.8% (24.1–61.3, *p* = 0.0004), respectively. Some adverse effects included gastroenteritis, pneumonia, and convulsions.
6	Mendoza et al. [33]	23 April 2019	Understanding the possible safety outcomes with the use of RTS,S/AS01in infants and children of sub-Saharan Africa	N = 8922 children (5–17 months), n = 6537 infants (6–12 weeks)	The study was trying to compare RTS,S/AS01(R3R) to the non-malaria control vaccine (C3C). During the 2–3-day period after the vaccine, there was an increase in febrile convulsions in the RTS,S/AS01 group compared with the control. The study also showed there were increased meningitis cases in children receiving RTS,S/AS01 (R3R: 11, R3C: 10, C3C: 1) but not infants. There was increased all-cause mortality in girls receiving RTS,S/AS01 (2.4% vs. 1.3%, all ages) compared with the C3C group.
7	Verma et al. [34]	4 March 2024	Looking at the effectiveness of the R21/Matrix-M malaria vaccine and RTS,S/AS01 (RTS,S) in children across 4 African countries	N = 4800 children	The study wanted to test if the R21/M-Matrix had a faster effect than the standard RTS,S/AS01. The study showed vaccine efficacy values for seasonal areas of 75% and 74% at 12 and 18 months, respectively. There was also a higher seasonal effectiveness in children within the 5-to-17-month range. After the one-year point, the anti-NANP antibody titers had doubled after administration of the third dose.
8	Ishola et al. [51]	29 October 2022	Study focuses on whether the asymptomatic malaria affects the immune response to the 2-dose Ad26.ZEBOV and MVA-BN-Filo Ebola vaccine regimen in malaria endemic countries like Sierra Leone	N = 587 patients, n = 188 adults, n = 399 children	The study was looking at the geometric mean concentrations (GMCs) after the first and second doses of the vaccine using ELISA. The geometric mean ratios using malaria-positive and malaria-negative GMCs were also done. After the first dose, malaria-positive children around 1 to 3 years old (age group-specific GMR, 0.56; 95% CI, 0.39–0.81) had lower GMCs compared to the malaria-negative children. After the second dose, there was a decreasing GMC trend in all groups with no age specific data (GMR, 0.82; 95% CI, 0.67–1.02).
9	Katureebe et al. [8]	8 November 2016	Researchers studies the malaria burden in 3 locations around Uganda after using insecticides and residual spray	Walukuba (n) = 42,833, Kihihi (n) = 28,790, and Nagongera (n) = 38,690	The study was looking at the test positivity rate (TPR), incidence of malaria (episodes per person per year (PPY)), and human biting rates (HBR) for the three regions. The two control methods used were (LLIN) and indoor residual spraying (IRS) of insecticides. After the 28-month period in Walukuba using LLIN, no changes were found in TPR (26.5% pre-intervention versus 26.2% post-intervention; aRR = 0.70, 95% CI 0.46–1.06, *p* = 0.09) or incidence (0.39 episodes PPY pre-intervention versus 0.20 post-intervention; adjusted rate ratio [aRR] = 1.02, 95% CI 0.36–2.91, *p* = 0.97). Following the 21-month period of LLIN in Kihihi, there were no changes in TPR (49.3% pre-intervention versus 45.9% post-intervention; aRR = 0.83, 95% 0.58–1.18, *p* = 0.30), but a reduction in malaria incidence (1.77 pre-intervention versus 1.89 post-intervention; aRR = 0.65, 95% CI 0.43–0.98, *p* = 0.04) was observed. After the 12-month LLIN period in Nagongera, there was no change in the malaria incidence (2.82 pre-intervention versus 3.28 post-intervention; aRR = 1.10, 95% 0.76–1.59, *p* = 0.60), but there was a decrease in TPR (45.3% pre-intervention versus 36.5% post-intervention; aRR = 0.82, 95% CI 0.76–0.88, *p* < 0.001)
10	Jongo et al. [36]	31 December 2020	The study observed the immune response for different dosages of the PfSPZ vaccine (*Plasmodium falciparum* [Pf] sporozoites [SPZ]) in Tanzania	N = 9 (age 18–45 years, assigned to 3 doses of 9 × 10^5^ PfSPZ or NS), N = 9 (age 18–45 years, assigned to 3 doses of 1.8 × 10^6^ PfSPZ or NS for Group 1b), N = 12 (age 18–45 years, assigned as infectivity controls)	The vaccine efficacy for the 9 × 10^5^ PfSPZ group during the 3rd or 11th week was 100% (*p* < 0.000l, Barnard test, 2-tailed). For the 1.8 × 10^6^ PfSPZ group, the vaccine efficacy at 7.5 weeks was around 33% (*p* = 0.028). The researchers also concluded that the time to first time parasitemia after the vaccine was longer in the PfSPZ groups compared with the control.
11	Emmanuel et al. [54]	13 April 2024	The study sought to understand the willingness of the caregivers under the age of 5 years to give malaria vaccines to their children in Nigeria	N = 347	The study showed a higher percentage of people that accepted the vaccine, with around 78.4% and around 21.6% rejecting it. Researchers had also found that people with post-secondary or higher certification were more willing to accept the vaccine, with values around 55.6%.
12	Adeleke et al. [11]	5 December 2022	The study wanted to explore sulfadoxine-pyrimethamine intermittent preventive treatment in infants (SP-IPTi) in Nigeria to prevent malaria.	N = 1379 patients	The study showed that SP-IPT was safe in infants. However, there was no difference in the 9-month risks of hospitalizations and fever compared with the control.
13	Draper et al. [21]	11 July 2018	The study observed different vaccines for malaria that have recently been used in the clinical setting.		The study found that higher levels of monoclonal antibodies against the NANP repeat region were strongly correlated with better vaccine efficacy, particularly in vaccines like RTS,S/AS01. The researchers have also concluded that IV administration of the PfSPZ vaccine had a stronger CD8+ response than the subcutaneous method.
14	Otieno et al. [30]	22 January 2020	Testing the safety and immune response of the RTS,S/AS01 malaria vaccine in HIV-infected children in sub-Saharan Africa	N = 15,459	The study consisted of 6537 infants (6–12 weeks) and 8922 children (5–17 months). The study reported the anti-circumsporozoite (CS) antibodies of the RTS,S/AS01 vaccine and comparator vaccine group. Each group had received 4 doses. The antibody levels for RTS,S/AS01 and comparator groups were 193.3 EU/mL and 491.5 EU/mL, respectively (*p* = 0.0001).

## Data Availability

Data are contained within the article.
